# Caregiver perceptions of children’s linear growth in Bangladesh: a qualitative analysis

**DOI:** 10.1017/S136898001700427X

**Published:** 2018-03-26

**Authors:** Muttaquina Hossain, Scott Ickes, Lauren Rice, Gaelen Ritter, Nurun Nahar Naila, Tasnia Zia, Baitun Nahar, Mustafa Mahfuz, Donna M Denno, Tahmeed Ahmed, Judd Walson

**Affiliations:** 1 Nutrition and Clinical Services Division, International Centre for Diarrheal Diseases Research, Bangladesh (icddr,b), Dhaka, Bangladesh; 2 University of Washington, Department of Health Services, Seattle, WA, USA; 3 University of Washington, Program in Nutritional Sciences, Seattle, WA, USA; 4 University of Washington, Department of Pediatrics, Seattle, WA, USA; 5 Childhood Acute Illness and Nutrition Network, Nairobi, Kenya; 6 University of Washington, Department of Global Health, Box 359931, 325 Ninth Avenue, Seattle, WA 98104, USA; 7 University of Washington, Departments of Medicine and Epidemiology, Seattle, WA, USA

**Keywords:** Stunting, Linear growth faltering, Maternal perceptions, Child nutrition, Bangladesh

## Abstract

**Objective:**

To understand caregivers’ perceptions of children’s linear growth and to identify the cultural meanings and perceptions of risk associated with poor height attainment.

**Design:**

Three investigators from Bangladesh conducted twelve focus group discussions.

**Setting:**

The study was conducted in rural and slum settings in Bangladesh.

**Subjects:**

Participants included mothers and alternative caregivers (*n* 81) who were recruited by household screening. No eligible, recruited subjects refused participation.

**Results:**

Caregivers reported limited experience with growth monitoring services from the health system. Caregivers mainly use visual cues and developmental milestones to understand if children are growing properly, and recognize that children normally experience both weight gain and linear growth with age. Mothers expressed concern over children’s malnutrition and short stature, but did not discuss children’s failure to attain a ‘growth potential’ or distinguish inherited short stature from stunting. Caregivers interpret the consequences of poor height attainment as primarily social and economic and cite few health risks.

**Conclusions:**

Linear growth interpretation is determined more by community norms than by guidance from nutrition programming or the health system. Interventions to prevent or reduce linear growth failure may be perceived to have limited value where appropriate linear growth in children is determined by comparison to peers and siblings. Such perceptions may be significant barriers to programmes addressing stunting prevention in settings where many children are stunted. Efforts to raise awareness about the risks of linear growth faltering may need to consider delivering messages to caregivers that emphasize the social and economic consequences of stunting.

Globally, 159 million children under 5 years of age suffer from linear growth failure or stunting (height-for-age *Z*-score<–2) of which South Asia alone accounts for 40 % of the total global burden^(^ ^1^ ^,^ ^2^ ^)^. On average, infants in South Asia are born 0·5 sd below global reference means, and linear growth deficits accumulate throughout the first 3 years, with the sharpest declines from birth to 6 months^(^ ^3^ ^)^. In Bangladesh, stunting rates have declined by 15 % from 2004 to 2015, but the burden remains high with a prevalence in 2015 of 36 %^(^ ^4^ ^)^.

The typical pattern of linear growth faltering begins *in utero* and progresses through early childhood^(^ ^3^ ^)^. Poor linear growth is associated with deficits in cognitive function, educational attainment, increased risk of chronic disease in adulthood and intergenerational growth deficits^(^ ^5^ ^,^ ^6^ ^)^. Within low- and middle-income countries, there is considerable variation in stunting between regions, within countries and between households^(^ ^7^ ^)^. A recent forty-five-country analysis demonstrated that stunting is more common in slum areas compared with non-slum areas (73 *v.* 24 %, respectively)^(^ ^8^ ^)^.

Multisectoral efforts to deliver interventions and programmes to the most vulnerable populations are needed in order to achieve the World Health Assembly goal of reducing stunting by 40 % by 2025^(^ ^2^ ^,^ ^9^ ^)^. While routine growth monitoring is part of a recommended set of strategies to prevent undernutrition, access to this basic health service is often limited in settings with underfinanced health systems. In addition, growth monitoring effectiveness is improved when coupled with quality counselling and referral to services that translate into the successful adoption of health behaviours by caregivers^(^ ^10^ ^)^.

Community and caregiver awareness about the importance of stunting, the signs of early growth faltering, and its long-term impacts on health and human capacity, are critical for effective programme development and delivery. Successful messaging and communication strategies require in-depth, contextually specific knowledge. Currently, there is a lack of understanding of the ways in which caregivers recognize and respond to children’s linear growth, especially in settings where poor height attainment may not be perceived as abnormal^(^ ^11^ ^)^. In settings with a high prevalence of stunting, caregivers may fail to recognize linear growth failure in children, despite an ability to perceive signs and symptoms of severe acute malnutrition^(^ ^12^ ^)^. In stunting-prevalent settings, mothers themselves are often stunted. Other factors, such as low literacy and depression, which are independently associated with growth faltering in their children, may also limit mothers’ abilities to appropriately understand growth monitoring information^(^ ^13^ ^–^ ^15^ ^)^.

To inform future interventions designed to prevent or reduce stunting, we conducted a qualitative study in urban and rural Bangladesh to (i) understand the signs that caregivers use to interpret their children’s linear growth and (ii) identify the cultural meaning associated with poor height attainment among children.

## Methods

### Study setting

One urban and one rural area were chosen for the present study to gain perspectives of caregivers in each context. The urban setting, Mirpur, is located in Dhaka and has a population of approximately 500 000 residents in an area of 14 km^2^ (35 000 residents/km^2^). This community is inhabited by low- and middle-income families and has conditions that are typical of a congested slum settlement^(^ ^16^ ^)^. The rural setting, Mirzapur, is located approximately 75 km from Dhaka and has a comparatively lower population density of 1091 residents/km^2^. Approximately half of the households have access to improved sanitation and 60 % have electricity. Men are mostly engaged in rice and jute production or daily wage labour, often abroad, whereas women work mainly in the home^(^ ^17^ ^)^. Focus group discussions (FGD) in both urban and rural settings were conducted in a field office by three study investigators (M.H., N.N.N., B.N.) who had previous experience in qualitative research. In addition, three research assistants consented participants, administered the sociodemographic questionnaire and took field notes during the FGD. These research assistants were postgraduates with backgrounds in sociology and anthropology (two female and one male), had advanced training in qualitative methods and had 3 years of experience in qualitative data collection procedures.

### Participants and recruitment

We adhered to the Consolidated Criteria for Reporting Qualitative Research (COREQ)^(^ ^18^ ^–^ ^20^ ^)^. The current study was nested within a larger study that examined caregiver perceptions of children’s growth and appetite. We conducted twelve FGD, with six FGD in the urban setting and six FGD in the rural setting. To investigate differences in how growth was understood by caregivers by child age and developmental stage, we organized three focus groups by child age: 6–12 months, 13–24 months and 25–59 months. Also, to understand the differences by caregiver experience, two focus groups at each site were composed; one consisting of experienced mothers who had more than one child and the other with first-time mothers. For groups involving alternative caregivers, participants were required to be an adult who provided full- or part-time care to a child aged 6–59 months who was either consuming complementary foods in addition to breast milk or was on a family diet. Fathers and paternal grandmothers were specifically recruited because they are known to be key decision makers for child health in this context^(^ ^21^ ^)^. The research team has a longstanding relationship with the study communities and maintains a household enumeration that includes demographic information. Using this register, we identified households in Mirpur and Mirzapur that contained participants who fit the eligibility criteria. Field staff visited eligible participants at home and explained the study objectives. Potential participants were asked if they would be willing to attend an FGD about caregiver experiences with understanding growth and appetite in young children. Mothers and alternative caregivers who expressed interest in participation were scheduled for interviews the following week. All eligible mothers or alternative caregivers in both settings who were approached for study enrolment agreed to participation. Each focus group consisted of six to eight mothers and/or alternative caregivers (*n* 81). The data collection was conducted between June 2016 and July 2016. The time and location of the discussions were confirmed with participants via telephone on the day before the FGD. Prior to each discussion, we briefed participants about the benefits, privacy and confidentiality of the study. After obtaining written informed consent and permission to audio record the discussion, research assistants administered a brief sociodemographic questionnaire to participants. Each FGD lasted approximately 90 min. Participants were provided with a financial incentive equivalent to $US 5 as compensation for their time and transportation costs.

### Conceptual framework and study tools

**Fig. 1:**
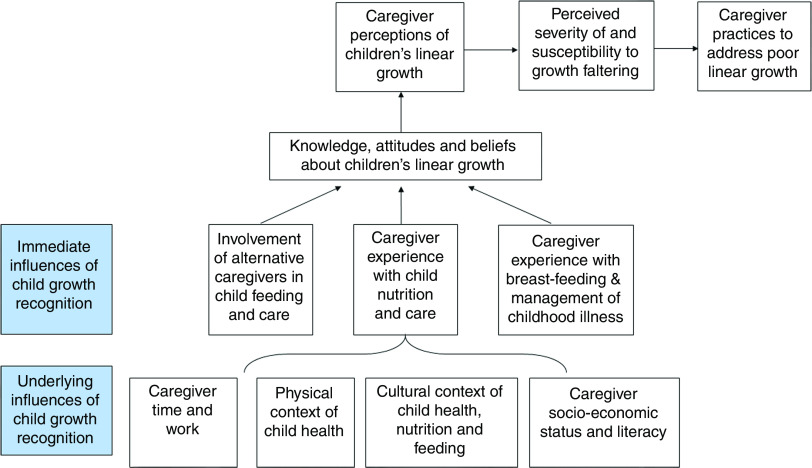
Conceptual framework of the proposed underlying and immediate influences on caregiver perceptions of children’s linear growth

Figure 1 presents the conceptual framework for the study. The framework is based on three models: (i) the Health Belief Model, with a focus on perceived severity of morbidity and mortality from poor growth and susceptibility to growth faltering and related problems; (ii) Bronfenbrenner’s social ecological model, which emphasizes the interplay of underlying and immediate influences of caregiver’s recognition of children’s linear growth; and (iii) the UNICEF framework of undernutrition^(^ ^22^ ^–^ ^24^ ^)^. Underlying influences include caregivers’ time utilization and work practices (such as maternity leave policies if employed, commuting time and the type of work), the physical context of child feeding (urban or rural) and the cultural context of child health, nutrition and feeding of young children (the culture of breast-feeding and complementary feeding, normative feeding styles, and caregiver concern over growth and thinness). Examples of immediate influences include whether grandmothers or aunts are the main participants in child feeding, the number of previous children cared for by a mother, and whether mothers have received education about child nutrition and have experience with treating sick children. These underlying and immediate factors are expected to exert influence on knowledge, attitudes and beliefs about children’s linear growth. Caregiver perceptions of linear growth in children are influenced by knowledge, attitudes and beliefs about linear growth, and by general caregiver capabilities, including responsive parenting^(^ ^25^ ^)^. Finally, caregiver perceptions of growth may influence the caregivers’ perceived susceptibility to child growth faltering and the severity of growth failure, which in turn will influence if and how they act based on their concerns.

The FGD guide (see online supplementary material) was developed from the conceptual framework to address the study objectives. The discussions were focused on caregiver perceptions of both child growth and appetite. We also investigated caregivers’ interactions with the health system related to children’s growth.

For the evaluation of perceptions of growth, participants were asked about caregivers’ understanding of whether a child’s height or length is appropriate, reasons why children may fail to grow properly and the potential consequences of poor linear growth. To centre the discussions on linear growth, the moderator was instructed to direct the conversation to caregiver awareness of children’s height/length. However, the discussions also included responses about weight gain as it is closely connected to linear growth. All study procedures were approved by the Institutional Review Board of the International Centre for Diarrheal Disease Research, Bangladesh (icddr,b). Written and verbal informed consent was obtained from all participants.

### Data analysis

FGD were audio recorded and transcribed verbatim into Bangla, and then translated into English by a bilingual speaker. Participants were noted in transcripts as P1, P2, etc., to link statements with demographic data. English transcripts were uploaded into Dedoose qualitative data analysis software (Los Angeles, CA, USA)^(^ ^26^ ^)^ and double-coded by three trained qualitative researchers. The coding team collectively developed the codebook based on the interview questionnaires (see online supplementary material) and from emergent findings. We developed a codebook that was continuously revised to ensure updated, consistent codes meanings and definitions.

The agreement was achieved in coding through discussion of codes after initial coding; the study team arbitrated any disagreements. We used the constant comparative method to analyse the interview transcripts, with an emphasis on comparing responses by the different participant groups^(^ ^27^ ^)^. Direct quotes were extracted from interviews and linked to the demographic data collected at the beginning of the FGD. Results were analysed according to ten codes that were developed deductively from the interview guides and inductively from study findings. These codes were: (i) caregiver’s general understanding of growth; (ii) signs and indications of healthy growth; (iii) physical/biological consequences of healthy growth; (iv) signs and indications of unhealthy growth; (v) physical/biological consequences of unhealthy growth; (vi) concern about a child’s height for age; (vii) child age and growth; (viii) child gender and growth; (ix) experience with formal health system/growth monitoring services; and (x) genetics/heritability. These results were then further aggregated into main topics.

## Results

Twelve FGD were conducted which included a total of eighty-one participants, with forty-one from the rural site and forty from the urban site. Table 1 summarizes the demographic characteristics of the study participants. Most of the participants were mothers (*n* 67, 82·7 %); the remaining participants were alternative caregivers (*n* 14, 17·3 %). Education levels were higher in the rural setting: 65 % of rural participants had any secondary school (grade 6 or higher) compared with 35 % of those in the urban setting. Mean age at first pregnancy was 2 years higher among rural mothers than their urban counterparts (20·8 *v.* 18·8 years). Over half of participants were first-time parents (55·2 %). Most mothers (88 % of sixty-seven mothers) were full-time homemakers. Urban mothers were more likely than rural mothers to be employed outside the home (21 *v.* 6 %). Findings were grouped into three main topics: (i) perceptions related to identifying and understanding children’s growth and growth failure; (ii) perceptions regarding the consequences of linear growth failure in children; and (iii) urban and rural differences (Table 2).

**Table 1:** Demographic characteristics of study participants: mothers and alternative caregivers (*n* 81) from rural and urban slum settings, Bangladesh, June–July 2016

	Rural (*n* 41)		Urban (*n* 40) Number	
	Mean or *n*	95 % CI or %	Mean or *n*	95 % CI or %
Maternal age (years)	26·6	24·1, 29·1	24·8	22·7, 26·8
Age <18 years	3	9	2	5
Age 18–35 years	27	88	30	75
Age >35 years	3	9	2	5
Alternative caregiver’s age (years)	46·5	40·3, 52·7	47·5	40·2, 54·7
Experience of mother (according to number of children)				
One child	19	58	18	53
More than one child	14	42	16	47
Maternal education				
No formal education	1	3	4	12
Any primary school (1–5th grade)	7	21	15	44
Any secondary school (6–10th grade)	21	64	12	35
Higher secondary school (11th grade or higher)	4	12	3	9
Maternal occupation				
Housewife, homemaker	31	94	27	79
Working formally outside the home	2	6	7	21
Parent’s age at first childbirth				
Mother (years)	20·8	–	18·8	–
Father (years)	29·0	–	24·6	–
Age of youngest child (months)	26·0	–	23·5	–

Data are presented as mean and 95 % CI for continuous variables, or as *n* and % for categorical variables.

**Table 2:** Result topics according to codes, definitions, and illustrative quotes from mothers and alternative caregivers (*n* 81) from rural and urban slum settings, Bangladesh, June–July 2016

Results topic	Code	Definition	Illustrative quote
Topic 1: Knowledge, attitudes and beliefs about healthy growth	Signs of healthy growth	Physical or biological signs of healthy linear growth	Physical signs:
			‘At first the weight increases and then the height. The child also eats more. Seeing these we understand. Then the baby laughs when spoken to and if I clap then he claps and when I call my baby “sona” he turns around and coos in contentment. The child’s weight increases and he plays and then his height increases – these things.’ (Age 22 years, one child, rural)
			‘When they complain that they are feeling pain in their hand and foot. Sometimes they complain of cramping pain in both limbs. At that time, I understand that, yes, they are growing properly.’ (Age 45 years, six children, urban)
			Developmental milestones:
			‘They try to talk and then want to play more and more. Try to stand up and walks around a lot. And then talks a lot, I mean listens to what we say. And then eats foods a little more. So these are the things.’ (Age 40 years, two children, rural)
			‘They will remain cheerful and play gleefully. They also feel pain when they are having growth.’ (Age 45 years, six children, urban)
Topic 2a: Knowledge, attitudes and beliefs about causes of growth failure	Signs of growth failure, physical	Physical or biological signs caregivers use to identify if children’s linear growth is inhibited	‘About my child, I would say he is less grown, weighs less. Because he remains small as a 3-year-old child. Doesn’t eat properly. Still reducing his weights instead of gaining.’ (Age 25 years, two children, rural)
	Signs of growth failure, other	Other signs that caregivers use to identify if children’s linear growth is inhibited	‘Well, when they are having insufficient amount of foods or refused to eat more foods, having less sleep then I realize that my child is not growing enough.’ (Age 19 years, one child, rural)
			‘Well, they are always running and playful. They play all the time. They do not stay well and that’s why they are not growing.’ (Age 38 years, alternative caregiver, rural)
Topic 2b: Knowledge, attitudes and beliefs about consequences of growth failure	Concern	Concern of caregivers over children’s growth failure	Social consequences:
			‘If a child remains short in stature it’s a matter of concern for the parents. Now in this era, everyone likes tall girls when it comes to marriage. These are the facts to worry.’ (Age 32 years, three children, rural)
			‘The problem is he will stand with other people, cannot get a job. He will feel inferior that all are tall but me … He may think I cannot be tall because of my parents or family. Depression and weakness.’ (Age 16 years, one child, rural)
			Biological consequences:
			‘If a child is weak and sick, has lack of nutrition then his intelligence will be less, which will lead him to failure in studying. Their brain works less.’ (Age 19 years, one child, urban)
Topic 3: Urban and rural differences	Urban *v*. rural	Difference in perceptions of growth faltering by study setting	‘Also, a child’s growth depends on their parents. If the parents are eating nutritious foods, then the children get nutrition too. Parents’ malnutrition causes a malnourished child.’ (Age 36 years, three children, urban)

### Topic 1: Perceptions related to identifying and understanding the causes of children’s growth and growth failure

#### Topic 1a: Perceptions related to identifying children’s growth and growth failure

When asked about the information used to indicate poor linear growth, mothers described four types of information: physical signs, eating patterns, developmental milestones and interactions with the health system.

*Physical signs*. While respondents linked poor height attainment to health outcomes (e.g. increased likelihood of illness), mothers provided a limited set of physical signs of healthy growth (e.g. expecting linear growth to increase with age, comparison with siblings and peers) that they used to determine whether their child experienced poor linear growth. As one mother noted:

‘Yes, when I compare with other children of same age and I see that they are taller and weigh more than my child, I understand that my child is not growing well.’ (Age 23 years, one child, urban)

Caregivers recognized that, as a child ages, weight gain coincides with linear growth. However, caregivers generally did not differentiate between linear growth failure and inadequate weight gain. Children’s weight was more commonly monitored than height and was used as a marker of healthy growth and the absence of illness:

‘In a normal sense, I understand his growth through his feedings, his weights. I measure his weight every month. I measure his weight whenever I can, to see whether he is growing up.’ (Age 50 years, alternative caregiver, urban)

Most caregivers had little experience with tracking children’s height and did not precisely understand appropriate height-for-age for their children. One experienced mother represented her group:

‘Actually, none of us knows how much in centimetres a child should grow in each month. I think none of the mothers knows this, that’s why we are having difficulty answering this.’ (Age 32 years, three children, rural)

While one mother noted that ‘perfect growth’ was defined as ‘if a child grows one inch in each month’, the general understanding of what was normal for a child was vague:

‘There is a certain height that a child should attain as his age progresses, which should increase steadily.’ (Age 27 years, two children, rural)

Some caregivers used other children to benchmark their own children’s growth:

‘If my 1-year-old child remains short of a [typical] year-old child, then that’s a problem.’ (Age 22 years, one child, urban)

In addition to direct height comparisons, caregivers expressed concern if children fit into clothing designed for younger children:

‘Suppose, today I dress him in clothes that become too short for him to wear later. I will realize that he is growing up. That’s how I realize that the child is getting taller.’ (Age 36 years, three children, urban)

While caregivers connected their child’s growth to a general reference to getting progressively taller and gaining weight, they did not frequently mention the concept of a child reaching his/her individual linear growth potential.

##### Eating patterns

Respondents indicated that healthy growth in children’s weight and height was a result of proper feeding:

‘Physical structure gives me the idea of whether the child is growing up or not … When a child doesn’t eat, loses appetite, gets sick his weight drops too. But when he is well and eats properly his physical structure develops and gets tall too.’ (Age 32 years, three children, rural)

Caregivers described greater intake in the amount and variety of foods as indicators of healthy growth. As one mother said:

‘When the child is eating vegetables, fruits, eggs, milk, fish, meats, I see that he is healthy and growing up.’ (Age 30 years, two children, rural)

Improvements in children’s appetite were also a noted indication of growth:

‘Now I see that [my child] eats rice two times and I understand that he is growing up. Because earlier I had to feed him forcefully, but now I do not have to do that. So, I understand that he is growing up.’ (Age 28 years, one child, rural)

Some participants had difficulty separating the concept of a healthy appetite and feeding from growth.

##### Developmental milestones

Caregivers, especially among mothers of children under 2 years of age, indicated that they interpreted children’s achievement of developmental milestones (e.g. ‘learning to hear’, ‘making observations around themselves’, talking and walking) to identify healthy growth. Weight gain rather than height gain was perceived as a more important indicator to them. Mothers described interactions with children and interpreted children’s responsiveness as a sign of healthy growth. As one adolescent mother noted:

‘My child understands me when I speak. For example, if I ask him something, he responds back to bring him something.’ (Age 15 years, one child, urban)

Children’s mimicking of caregivers’ gestures also indicated growth:

‘When I clap my hands, he also responds back clapping his hands. When I play with him, he responds back cheerfully.’ (Age 22 years, one child, rural)

##### Interaction with the health-care system

Caregivers described having limited interaction with formal growth monitoring services. Consultation with health professionals was mentioned only by rural participants. Rural caregivers noted that they seek medical advice if encouraged by their families in cases when child illness is of concern:

‘We have to go to the doctor to ask why the child is not growing. It’s not a problem if a child is short, but everyone in the family says that it looks better when a child is tall. It feels good that when I am feeding my child and he is growing up. If he is not growing tall, then I have to take advice from the doctor for why he is not growing up.’ (Age 19 years, one child, rural)

While most caregivers did not describe actively seeking guidance or assessment from health professionals, some noted that such advice is valuable:

‘We have to take advice from people like health workers, like doctors from the health centre, about the importance of children’s growth, because a lot of importance should be given to a child’s growth.’ (Age 34 years, one child, urban)

Main findings from Topic 1a are presented in Box 1.

Box 1 Main findings from Topic 1a (perceptions related to identifying children’s growth and growth failure)

**1.** Caregivers in both the urban and rural groups identified a small number of physical signs used to recognize healthy growth. Caregivers mainly used physical indicators such as a child’s height and weight to assess healthy growth, and compared their children’s heights and weights with those other children of the same age. Height and weight were not typically measured, but more likely to be visually perceived and monitored to see if children were progressing in terms of length/height and weight.

**2.** Caregivers looked for developmental milestones in children to identify healthy ponderal and/or linear growth. For most mothers, these centred around alertness and learning.

**3.** Caregivers described a greater intake in the amount and variety of foods as indicators of healthy growth.

#### Topic 1b: Perceptions related to understanding the causes of children’s growth and growth failure

##### Genetics/heredity

Most caregivers cited hereditary factors when asked about the reasons behind poor height gain. They noted that children’s height will reflect their parents’ height:

‘Well, suppose height is a matter related to the family. If everyone in the family is short, then the child will be short too. If everyone in the family is tall, then the child will be tall too.’ (Age 16 years, one child, rural)

Caregivers described that short parents expect to have short children, which does not reflect unhealthy growth:

‘Usually it [not growing tall] is said for genetic reasons. And if it is genetic, it is not a problem.’ (Age 16 years, one child, rural)

*Illness*. Some caregivers noted that their children might suffer from a ‘hidden illness’, resulting in stunting and growth failure:

‘If the child doesn’t eat properly and grow well and remains short, then we assume that this child must have some problems. That’s why he is not growing up.’ (Age 23 years, one child, urban)

Caregivers made different connections between height attainment and body weight. One urban mother (aged 16 years) showed concerns of being obese in future due to short stature during childhood, while an older rural mother (aged 37 years) stated that short children ‘become very skinny’ and grow weak. Taller children were thought to become ill less often; illness, in turn, made growth more difficult:

‘If a child remains sick all the time, he will not grow. Their body will be thin, and they will stay short.’ (Age 24 years, one child, urban)

Main findings from Topic 1b are presented in Box 2.

Box 2 Main findings from Topic 1b (perceptions related to understanding the causes of children’s growth and growth failure)

**1.** Caregivers often recognized that genetics/heredity may explain why children are short; therefore, if parents are short and their child is short, this may not indicate unhealthy growth.

**2.** Even when children eat nutritious foods, illness may result in stunting and growth failure.

**3.** Caregivers relied on visual perceptions of children’s physical appearance to determine appropriateness of growth according to age; weight, more than height, is used to indicate undernutrition.

**4.** Caregivers described little if any interaction with specific growth monitoring services, and did not cite the health system as a commonly accessed source of information regarding appropriate growth.

#### Topic 2: Perceptions of the consequences of linear growth failure in children

##### Social and economic consequences

Some caregivers expressed substantial concern over linear growth failure and recognized that:

‘… we have to take advice from people like health workers, doctors from health complex about the importance of child’s growth because a lot of importance should be given on child’s growth.’ (Age 24 years, two children, urban)

Some caregivers believed that a child’s height, weight and brain are very important because ‘intelligence, thinking power, height, and weight all are related’ and that a child’s poor height attainment can result in diminished mental health (age 50 years, alternative caregiver, urban). Most caregivers expressed social consequences, but not health consequences, associated with unhealthy growth. The social and economic consequences cited by caregivers included poor education and job attainment, decreased marriage prospects, reduced social acceptance and feelings of isolation:

‘It’s a worry because they cannot enter into jobs. They cannot fit in anywhere and they remain unemployed.’ (Age 25 years, two children, rural)

##### Disagreement over the ‘consequences’ of short linear growth

While most caregivers agreed that there were economic and social consequences of being short, a subset of the caregivers disagreed and felt that there are no economic and social consequences derived from short stature. Some caregivers cited that ‘it’s up to God’ to decide how tall a child should be and that:

‘… short people can get jobs too. Those who studies, are intelligent and get jobs as well.’ (Age 40 years, alternative caregiver, urban)

Main findings from Topic 2 are presented in Box 3.

Box 3 Main findings from Topic 2 (perceptions of the consequences of linear growth failure in children)

**1.** Caregivers cited a variety of consequences of improper growth, from poor education and job attainment to decreased marriage prospects, the latter which was a concern, especially for girls. Some caregivers worried that even with good education, short children will have trouble being accepted in the society.

**2.** Both urban and rural caregivers cited social and economic consequences of poor linear growth as more important than health consequences; however, caregivers disagreed over the specific social and economic consequences of being short.

**3.** Urban caregivers drew more specific connections between nutrition during pregnancy and children’s growth, and were more likely to note the relationship between poor growth in childhood and eventual adverse pregnancy outcomes when those children became mothers.

#### Topic 3: Urban and rural differences

Despite many similarities between settings, urban caregivers demonstrated a greater understanding of the consequences of poor growth. First, urban caregivers were more likely to mention developmental milestones as indicators of growth, such as the ability of children to recognize parents, the desire to play and the ability to learn to talk. Second, urban, but not rural, caregivers drew a connection between parental nutrition and child growth:

‘Also, a child’s growth depends on their parents. If the parents are eating nutritious foods, then the children get nutrition too. Parents’ malnutrition causes a malnourished child.’ (Age 36 years, three children, urban)

Finally, urban caregivers drew connections between being short in childhood and having future problems with childbirth, noting that

‘… short women face problems when they get pregnant.’ (Age 30 years, three children, urban)

## Discussion

To our knowledge, the present study is the first to investigate caregiver perceptions regarding children’s linear growth. In Bangladesh, where approximately half of children under 5 years old from poor households are stunted, understanding perceptions of stunting among caregivers in an important step in identifying interventional strategies to reduce or prevent stunting^(^ ^4^ ^)^. These data suggest that the highly prevalent nature of stunting in Bangladesh may directly contribute to difficulties experienced by caregivers in identifying linear growth failure and in appreciating the potential adverse health consequences associated with stunting. Promotion of strategies to address some of the information gaps in the recognition of linear growth failure and its consequences, particularly among caregivers with lower literacy, may be important to improve linear growth outcomes^(^ ^15^ ^)^.

Efforts to prevent or reduce stunting among children are being scaled up globally, with an emphasis on multisectoral initiatives to address the underlying factors that influence poor linear growth^(^ ^28^ ^)^. The Government of Bangladesh has made commitments to reduce stunting and has approved a new national nutrition policy that has a focus on stunting reduction to achieve the sustainable development goal target for stunting^(^ ^29^ ^)^. Efforts to raise caregivers’ awareness of stunting may offer an opportunity to improve understanding and optimize interventional effectiveness by improving acceptance and adherence. The design of communication strategies to improve caregiver awareness requires a detailed understanding of when and how caregivers recognize children’s linear growth failure and how they interpret potential adverse consequences.

In the current study, caregivers reported variable experience with accessing growth monitoring and promotion (GMP) services and did not consistently cite GMP services as a major source of information to understand children’s normal linear growth. Multiple studies across a variety of settings with a high burden of undernutrition document low participation in GMP programmes^(^ ^30^ ^–^ ^32^ ^)^. Caregivers may elect not to participate in growth monitoring services where the benefits of formal growth evaluation are not clear enough to encourage attendance^(^ ^31^ ^,^ ^33^ ^)^. For example, mothers in Ethiopia cited lack of supplementary feeding at GMP sessions and a lack of perceived illness in children as the two leading causes of not utilizing GMP services^(^ ^30^ ^,^ ^32^ ^)^. Despite global efforts to shift the focus of GMP from a curative to preventive focus, a study of district medical officers from multiple countries reported that caregivers more often use GMP services after a health problem is identified, rather than as a prevention tool^(^ ^31^ ^,^ ^33^ ^)^. In Bangladesh, large-scale GMP services have been offered for over a decade^(^ ^10^ ^,^ ^34^ ^)^. In the absence of illness of the child, caregivers may have difficulty in prioritizing attendance at growth monitoring sessions. In addition, GMP has been identified to be a more successful strategy to promote healthy growth if linked to effective nutrition, health and counselling services^(^ ^34^ ^,^ ^35^ ^)^. Community-based approaches may represent a platform for such GMP, given the success of community health workers in promoting other maternal and child health services in Bangladesh, such as care provision from the 18 000 community clinics established throughout the country^(^ ^36^ ^)^.

Low maternal education and lack of awareness of proper feeding practices among mothers make maternal engagement with growth monitoring and counselling more difficult^(^ ^32^ ^,^ ^37^ ^,^ ^38^ ^)^. Caregivers in the rural context expressed greater concern about their children’s growth and reported accessing GMP services more frequently than their urban counterparts. Higher maternal education levels in the rural setting may be responsible for some of this difference. Caregivers living in the slum context reported having comparatively lower access to health services and were generally more disadvantaged than those living in rural settings. Thus, this group may be particularly vulnerable and may benefit from programmes aimed at increasing growth awareness and ameliorating growth^(^ ^39^ ^)^.

Our findings suggest that caregivers lack appropriate biomedical knowledge regarding child’s linear growth trajectories, instead relying on personal instincts and peer comparisons to understand children’s height attainment. While global consensus exists regarding how to define and measure stunting in children^(^ ^40^ ^)^, coupled with growing international advocacy efforts to prevent stunting, the problem often goes unrecognized in communities where short statue is normative and where linear growth is not assessed in the primary health-care system^(^ ^41^ ^)^.

### Limitations

There are several limitations to this analysis. The study design enabled the identification of main themes by participant group, but does not allow us to express the frequency of these themes or to rank order them according to the level of importance. Second, the analysis is limited to the inclusion of a limited number of categories of focus groups (first-time mothers, mothers of children under 1 year, alternative caregivers). In addition, while we attempted to develop broad interview guides that could elicit responses that may be applied elsewhere, the cultural context of the study may not translate to other settings.

## Conclusions

The present study suggests that the interpretation of linear growth and linear growth faltering among Bangladeshi caregivers is determined more by community norms than by guidance from nutrition programming or the health system. Caregivers frequently noted that the absence of illness was an indication of growth and often did not appear to appreciate the need for children to meet a biologically defined growth potential. Finally, the practice of assessing the appropriateness of children’s linear growth by comparing children with peers and siblings, without comparison to a normative growth standard, is problematic in settings where many children are stunted. Communication of the risks of linear growth faltering in a population where stunting is highly prevalent may require novel information, education and communication strategies to effectively reach caregivers regarding the importance of identifying children with linear growth faltering and the prevention of linear growth failure^(^ ^42^ ^)^. In addition to messaging about health risks of stunting, efforts to raise awareness about the risks of stunting in the Bangladeshi context may be able to effectively leverage concerns about social and economic consequences of poor height attainment, as these outcomes were more salient to caregivers.

## Supplementary material

For supplementary material accompanying this paper visit https://doi.org/10.1017/S136898001700427X. click here to view supplementary material
