# Supplementary material for: Caregiver perceptions of children’s linear growth in Bangladesh: a qualitative analysis
Source: Public Health Nutr. 2018 Mar 26;21(10):1800–9. doi: 10.1017/S136898001700427X (PMC6088532; doi:10.1017/S136898001700427X)
Supplement: Supplementary file 1 [file S136898001700427Xsup001.docx]

**Note: Domain 4 was the main question set used for this analysis of the article “Caregiver perceptions regarding linear growth of children in Bangladesh: a qualitative study.”**

**Study aims and approach**

**Aim 1**: To examine maternal understanding of children's appetite during both healthy and non-healthy periods.

Approach: We will explore commonly used words, phrases, perceptions visual signs, emotional, and behavioral information that caregivers process to determine if children have an appetite. We will also assess what major maternal factors may influence how appetite is understood.

**Aim 2**: To describe caregivers’ perceptions of stunting as a condition of child malnutrition or illness in Bangladesh.

Approach: We will examine if and how caregivers recognize growth failure in Bangladeshi children through understanding the visual, physical, emotional, and behavioral signs that caregivers use to assess if children are growing adequately, with a focus on linear growth.

*********

**Explanation of purpose**

“Thank you for participating in today’s discussion. The purpose of today’s discussion is to learn from you about how caregivers in Bangladesh recognize appetite in children under 5 years old. We also want to know what information mother’s use to understand if their child is growing well. Everyone here has experience with mothering and feeding young children, and we appreciate the wisdom that you will share in our discussion today.”

| **Domain 1: Words and phrases used to describe appetite.** |  |
| --- | --- |
| 1. Tell me the words and phrases that you use to describe if their children have an appetite. |  |
| - 1. If you were to describe if your child has appetite, how do you describe it?   *(Note: if an example is needed, we can say “hungry”)* |  |
| - 1. Are there any other words or phrases used by other caregivers in your community used to describe appetite?   *Probe*: How do other caregivers describe appetite?  *Note: Try to note type of caregiver (e.g., mother-in-laws, fathers)* |  |
| 1. Tell me about all of the words and phrases that caregivers use to describe if a child does **not** have an appetite*(/is not hungry).* By not having an appetite, I do not mean because they are full, but rather because of illness or problem that causes them to not want to eat. |  |
| **Domain 2: Physical and emotional cues/signs/symptoms used to describe appetite.** |  |
| 1. Tell me the cues that tell you if your child has an appetite. How does your child behave when he or she has an appetite?   *Note: Looking for symptoms, emotions, and behaviors (e.g. pointing, rooting, crying, fussing).* |  |
| - 1. If you were to notice your child has appetite, what would you look for to notice it? First, think about young babies under 6 months, then 6 to 24 months, then 2-5 year olds. |  |
| - 1. Are there any other signs used by other caregivers *(e.g., other mothers, aunties, fathers, mother-in-laws)* to identify if a child has an appetite? |  |
| 1. a. Now please tell me about the cues that tell you and other caregivers if your child does **not** have appetite. By not having an appetite, I do not mean because they are full, but rather because of illness or problem that causes them to not want to eat. First let’s talk about newborn babies through 5 months, then 6 to 24 months, then 2 to 5 years.   *Note: Not about satiety but about potentially problematic or pathologic lack of appetite.* |  |
| b. Are there any other signs used by **other caregivers** to identify if a child does not have an appetite? Again, I do not mean because they are full, but rather because of illness or problem that causes them to not want to eat. |  |
| 1. How do caregivers know when X (*use the most common cues identified, e.g. crying, pointing, fussing*)? means that a child has an appetite or lack of appetite instead of doing those things for another reasons (e.g. being *hurt, sad*). |  |
| 1. We’ve talked quite a bit about how you can tell if your child has appetite. Are there other ways that you (or others) know when a child does or does not have an appetite? |  |
| 1. Think back to the last time your child was ill with something like diarrhea, or a fever. During that time, how were you able to tell if your child had appetite?   (*Try to probe the age as well)*  *Probe: Do caregivers look for anything different if a child is ill?* |  |
| **Domain 3: Maternal factors that influence awareness of cues** |  |
| 1. What are factors or reasons that mothers and other caregivers are not aware of the cues that we just discussed (*list them*)?   *Probe*: For example, do moms with less education or family support understand appetite differently? |  |
| **Domain 4: Caregiver awareness of child growth** |  |
| Finally, we want to talk about how caregivers recognize how well their children are growing. |  |
| 1. What are some signs that mothers and other caregivers look for to understand if their children are growing properly?   *Probe*: Is there anything else that mothers and other caregivers look at in children to understand if a child is not growing properly/is malnourished? |  |
| 1. Tell me how important a child’s height as a sign of how well the child is growing.   *Probe*: Are mothers and other caregivers ever concerned if a child is too short for his/her age? Why or why not?  *Probe:* *Does this differ by child’s age, gender, birth order?* |  |

This concludes our focus group. Thank you very much for your time and insights! We appreciate all that you had to say. Please remember to keep the things that we discussed today confidential.
